# Application of the solvent effect on bioluminescent reporter bacteria as a real-time membrane toxicity assay

**DOI:** 10.1099/acmi.0.001096.v3

**Published:** 2026-01-08

**Authors:** Phillip R. Myer, Ronald F. Turco, Bruce M. Applegate

**Affiliations:** 1 Department of Animal Science, University of Tennessee, Knoxville, TN, USA; 2 Department of Agronomy, Purdue University, West Lafayette, IN, USA; 3 Department of Food Science, Purdue University, West Lafayette, IN, USA

**Keywords:** Bioluminescence, bioreporter, *Pseudomonas fluorescens*, solvent

## Abstract

Bioluminescent bioreporters are widely used across various scientific disciplines due to the well-characterized bacterial bioluminescence mechanism. However, solvent-induced membrane perturbations may confound the use of bioreporters in assessing cellular toxicity from environmental contaminants. This study investigated the solvent effect, wherein membrane damage increases intracellular availability of bioluminescent reaction precursors, increasing the light produced. A new online *in situ* monitoring system was also tested with multiple bioluminescent reporters, including a newly constructed *Pseudomonas fluorescens* M3A strain, exposed to toluene, trichloroethylene, acetone, phenol and creosote derived from beechwood tar. Additional tests included the introduction of carbon nanotubes, fullerene and fullerenol. A solvent effect was confirmed by the detection of increased bioluminescent signal and the occurrence of fatty acid release (*P*<0.05). Phenol (25 p.p.m.), a benchmark for bactericidal activity, demonstrated luminescence enhancement via the solvent effect. Membrane toxicity assays showed that *P. fluorescens* M3A responded sensitively to sublethal and lethal membrane disruptions, whereas *Vibrio fischeri* MJ1 did not exhibit a solvent effect, and its luminescence changes were not correlated with viability (*P*>0.05). These results indicate that *P. fluorescens* M3A is a sensitive biosensor for detecting environmental contaminants and identifying both lethal and sublethal membrane perturbations. The findings underscore essential considerations when utilizing bacterial bioluminescence as a proxy for gene expression or cellular physiology.

**Impact Statement**

This study introduces an important refinement to the use of bacterial bioluminescent biosensors by characterizing the solvent effect as a confounding factor in real-time toxicity assays. By demonstrating that membrane-perturbing solvents enhance light emission independently of gene induction, the research challenges assumptions that bioluminescence directly reflects cellular viability or metabolic activity. The newly developed *Pseudomonas fluorescens* M3A strain proved highly sensitive to both lethal and sublethal membrane disruptions, enabling detection of toxicity well before conventional culture-based methods. Furthermore, the implementation of an online, *in situ* monitoring system allowed real-time quantification of membrane toxicity dynamics within seconds of exposure. This work is broadly relevant to environmental microbiology, toxicology, nanotechnology and biosensor development, particularly in contexts where rapid screening of environmental contaminants or industrial chemicals is essential. It provides a step forward in understanding the mechanisms that govern biosensor signal responses, emphasizing the importance of membrane integrity and substrate availability. The findings offer both an incremental and practical advancement by improving the interpretability and utility of bioluminescent biosensors in research and environmental monitoring.

## Data Summary

All supporting data are provided in the supplementary materials, including figures illustrating the lux biochemical pathway (Fig. S1), the configuration of the *in situ* bioluminescent detection system (Fig. S2) and results from nanoparticle light quenching analyses (Fig. S3A–C). These data support key findings on the role of substrate availability, membrane perturbation and nanoparticle interference in modulating bioluminescent signals. The authors confirm that all supporting data have been provided within the article or through supplementary data files.

## Introduction

Bacterial bioluminescence has long been utilized in biosensor design, where genetic reporters are combined with transducers to achieve functional and specific activity [1,3]. Engineered strains have facilitated the detection of heavy metals in wastewater [4], nanoparticle toxicity in soils [5] and antimicrobial efficacy [6,7]. Bioluminescent biosensors typically produce either a ‘lights off’ or ‘lights on’ response. For example, placing lux reporter genes under the control of inducible operons allows signal correlation with specific stimuli. This strategy has been employed in detecting DNA damage via *recA–lux* fusions [8], fuel contamination [3] and iron bioavailability [9]. Bioluminescence is often positively correlated with the concentration of the inducer, making it a proper quantitative signal.

While biosensors effectively assess toxicity, sublethal concentrations may still elicit physiological responses. Previous research suggests that solvents such as toluene and acetone, at sublethal concentrations, can augment bioluminescent signals [3]. This phenomenon, termed the solvent effect, occurs due to membrane perturbation increasing aldehyde precursor availability, thereby enhancing light production. Such nonspecific signal increases may confound the interpretation of quantitative data.

Conventional assays, like Microtox (AZUR Environmental, Newark, DE), rely on inverse correlations between luminescence and toxicity [10,12]. Differentiating between lethal and sublethal effects is crucial for understanding how contaminants interact with cellular mechanisms. This study examines the solvent effect across multiple bioluminescent reporter organisms using real-time monitoring and membrane toxicity assays, intending to refine the sensitivity of bioluminescent biosensors.

## Methods

### Bacterial strain maintenance and storage

All bacterial strains used in this study were maintained under standardized growth and storage conditions to ensure consistent physiological status prior to experimentation. Unless otherwise noted, *Pseudomonas* and *Escherichia coli* reporter strains were routinely cultured on Luria–Bertani (LB) agar plates supplemented with the appropriate antibiotic selection (kanamycin, 500 mg l^−1^ for lux-containing constructs). *Vibrio* strains were maintained on marine agar (Becton Dickinson, Franklin Lakes, NJ). Working cultures were generated from single colonies and grown overnight in their respective broth media at 26 °C with agitation (200–250 r.p.m.). Long-term storage stocks were preserved at –80 °C in sterile glycerol (final concentration 20%) and revived by streaking onto selective agar plates followed by overnight incubation. Prior to all experiments, cultures were grown to mid-exponential phase and adjusted to the appropriate optical density (OD_600_=0.35) as described in subsequent sections to ensure uniform metabolic activity and bioluminescent baseline levels.

### *Pseudomonas fluorescens* M3A construction

As described previously [13], *Pseudomonas fluorescens* Migula (ATCC number: 27663) was biparentally mated with an *E. coli* strain harbouring the *miniTn5 nahRG-luxCDABE*, carrying a kanamycin resistance gene, and the associated delivery vector [2,3]. Various ratios of a donor strain culture and recipient cultures were used in a tube-mating protocol [14]. The matings were allowed to proceed for 24 h, followed by plating on the appropriate selective solid medium. *P. fluorescens* matings were initially screened on *Pseudomonas* isolation agar to select against the *E. coli* donor, containing 1,500 mg l^−1^ kanamycin. The matings were serially diluted and plated. Ten isolates were subsequently subcultured onto LB (10% tryptone, 5% yeast extract, 10% NaCl, 1.7% agar) plates containing 500 mg l^−1^ kanamycin and 50 mg l^−1^ salicylate (inducer). One isolate produced visible bioluminescence after 24 h. The isolate, designated M3A, and the parent *P. fluorescens* were grown in minimal medium with succinate as a sole carbon and energy source to observe if the transposon had disrupted a key metabolic gene. Both the recombinant strain and the wild-type *P. fluorescens* strain exhibited similar growth rates. Cells were plated from the minimal medium onto LB agar plates containing 200 mg l^−1^ kanamycin and 50 mg l^−1^ salicylate. After 24 h of incubation, visible bioluminescence was observed from colonies.

### Solvent/toxin preparation

Concentrations used in this study were based on previously published solvent toxicity thresholds in bioluminescent reporter systems [3,10, 12, 15]. Aqueous solutions saturated with each solvent (toluene, phenol and trichloroethylene) were prepared by the addition of 1, 3 and 1 ml, respectively, of the solvent to 20 ml sterile water in a 25-ml mineralization vial and mixed on a rotary shaker (Barnstead Lab-Line MaxQ 2000) at 200 r.p.m. for 24 h at room temperature. After phase separation, aliquots of the aqueous phase were transferred to additional mineralization vials for dilution. Different volumes were used solely to ensure saturation based on solvent solubility differences and that only the aqueous phase was used for all subsequent dilutions and assays. Dilutions were prepared to obtain the following concentrations: toluene, 128 mg l^−1^; phenol, 50 p.p.m.; and trichloroethylene (TCE), 50 p.p.m. (based on water solubility). For acetone, a 1% (v/v) solution was prepared in sterile water and then diluted to obtain 1,960 mg l^−1^ of acetone. Creosote from beechwood tar (CAS number 8021-39-4) was prepared by the addition of 1 ml of the solvent to 20 ml of sterile water and mixed on a rotary shaker at 200 r.p.m. The water-soluble components of creosote were diluted in sterile water to obtain varying dilutions of the water-soluble components.

As-produced single-walled nanotubes (AP-SWNTs) were suspended in sterile water using sonication (1 h) at 0.5 mg ml^−1^ [5]. Mixed AP-SWNTs were then diluted to 215 µg ml^−1^. Considering the impurity and functional group content, the actual concentration of AP-SWNTs was 7.5 µg ml^−1^. Fullerenes, prepared and suspended with tetrahydrofuran, were diluted in sterile water to 50 p.p.m., while fullerenol was prepared by the addition of the nanomaterial to sterile water in a 25-ml mineralization vial and mixed on a rotary shaker at 200 r.p.m. for 24 h to obtain 50 p.m. Details on nanoparticle light quenching are provided in Page S4 (available in the online Supplementary Material).

### Bioluminescent detection of the solvent effect

Assays were conducted as described by [3,3]. Briefly, cultures from frozen stock were grown in 100 ml yeast extract peptone glucose (YEPG) broth (1% glucose, 2% polypeptone, 0.2% yeast extract, 0.2% NH4NO3) with the appropriate antibiotic selection [16]. After 20–24 h, a subculture was prepared at an optical density at 600 nm (OD_600_) of 0.35, and 2 ml aliquots of the exponentially growing culture were added to 25-ml EPA vials with Teflon-lined screw caps containing 2 ml of the solvent to be tested. Experiments were conducted at 25 °C with shaking at 300 r.p.m. on a Barnstead Lab-Line A-Class Max 200 orbital shaker. Bioluminescence and optical density (OD_600_) were monitored after an incubation time of 30 min using a Zylux luminometer (Zylux Corp., Huntsville, AL) and an Eppendorf Biophotometer (Eppendorf, Germany).

Since the inducible strains produce and recycle a fatty aldehyde substrate for the light reaction, the aldehyde may be rate-limiting [3]. Appropriately, where indicated, aldehyde (*n*-decanal) was added to saturate the bioluminescent system for control purposes. This was performed by adding 160 µl of a 1% (v/v) aqueous solution of *n*-decanal to the test sample prior to the light reading. Cell density was determined and used to normalize the bioluminescence values. Details on the characteristics of *P. fluorescens* M3A in differing media are provided in the Supplementary File.

### Membrane lipid fatty acid analysis

The quantification of free fatty acids within the experimental samples was performed using a free fatty acid quantification kit (Abcam, Cambridge, MA). After solvent treatment, samples were centrifuged at 10,000 ***g*** for 5 min on an Eppendorf 5415D Centrifuge (Eppendorf, Germany). The supernatant was then transferred to a new 1.5-ml microfuge tube, and the sample was concentrated using a miVac DNA Concentrator (Genevac Ltd, Stone Ridge, NY) at 45 °C for 20 min. Fatty acids were quantified via fluorescence (Ex/Em=535/590 nm) using a SpectraMax Gemini EM Fluorescence Microplate Reader (Molecular Devices, Sunnyvale, California), and the results were applied to a palmitic acid standard curve.

### *In situ* monitoring of the solvent effect

The assays were conducted as described earlier [3]. Cultures of *P. fluorescens* M3A from frozen stock were grown in 100 ml YEPG medium [16] with kanamycin (50 mg ml^−1^) and supplemented with 10 ml phosphate buffer (0.5 M, pH 7). After 20–24 h, a subculture was prepared with an optical density at 600 nm (OD_600_) of 0.35, and 2 ml aliquots of the exponentially growing culture were added to 25-ml EPA vials with Teflon-lined screw caps containing a magnetic stir bar. Light readings were monitored through a light pipe placed between the EPA vial and a photomultiplier tube (Oriel model 7734) connected to a PC running customized capturing software [13]. The vial was stirred with a Teflon-coated magnetic stir bar while in the light-tight chamber. A tube projecting out from the chamber and into the vial was used to inject 2 ml of the experimental solutions, as described by [13,13]. Readings were taken every second over a period of 15 min.

### Membrane toxicity assay

The membrane toxicity assays were conducted in a manner like the procedures mentioned above. *P. fluorescens* M3A cultures were grown overnight in LB broth at 26 °C and supplemented with kanamycin (50 mg l^−1^) and salicylate (50 mg l^−1^). A 1 ml aliquot of the overnight culture was then centrifuged at 15,000 ***g*** for 5 min using an Eppendorf 5415D centrifuge and resuspended in 1 ml of PBS (pH 7). This wash was repeated three times. The resulting culture was used to inoculate YEPG medium [16], supplemented with kanamycin (50 mg l^−1^) and phosphate buffer (0.5 M, pH 7). At an optical density at 600 nm (OD_600_) of 0.35, 2 ml aliquots of the exponentially growing culture were added to 25-ml EPA vials with Teflon-lined screw caps containing 2 ml of diluted creosote solutions, prepared in sterile water. Experiments were conducted at 25 °C in triplicate with shaking at 300 r.p.m. on a Barnstead Lab-Line A-Class Max 200 orbital shaker. Bioluminescence and OD_600_ were immediately monitored using a Zylux luminometer and an Eppendorf Biophotometer after a 30-min incubation. The sample was then serially diluted in PBS and plated on LB agar supplemented with kanamycin (50 mg l^−1^) and incubated at 26 °C overnight to determine cell counts.

As *Vibrio fischeri* MJ1 is utilized in the Microtox assay (AZUR Environmental, Newark, DE) as a bioluminescent reporter, which uses phenol as a standard, the assay was also conducted with *V. fischeri* MJ1. *V. fischeri* MJ1 cultures were grown overnight in marine broth (Becton Dickinson, Franklin Lakes, New Jersey) at 26 °C. The assay sample was also serially diluted in PBS, plated on LB agar and incubated at 26 °C overnight to determine cell counts.

### Data analyses

As with many real-time bioluminescent reporter systems, this platform functions as a single-sample continuous kinetic assay in which replicates cannot be run simultaneously; independent runs confirm reproducible kinetic trends, but variation in absolute luminescence values precludes meaningful point-wise error bars. Where appropriate and aside from analyses performed with the online *in situ* monitoring system, statistical significance of the results was evaluated using one-way ANOVA. When significant differences were found, post hoc comparisons were conducted using Tukey’s honestly significant difference test with a significance level set at *α*=0.05. Differences were considered statistically significant if the *P* value was less than 0.05. All statistical analyses were carried out using SPSS version 22.0.

## Results and discussion

### Bioluminescent detection of the solvent effect

A summary of the metabolic capabilities and genotypes of the bacterial strains tested for their light-generating abilities is presented in Table 1. To determine and establish the phenomenon of the solvent effect within *luxCDABE*-based bioreporter organisms, these organisms were challenged with sublethal concentrations of the solvents toluene (64 mg l^−1^) and acetone (980 mg l^−1^) [17]. We hypothesized that the availability of substrates, such as fatty aldehydes and fatty acids, would affect the bioluminescent response to the two solvents. Organisms were grown in YEPG and were not induced to produce light unless the *lux* cassette was constitutively expressed, i.e. *Salmonella poona* lux, *E. coli* K12 lux and *E. coli* O157:H7 lux (Table 1).

**Table 1.** List of *luxCDABE* bioreporter organisms used in the study

Organism	Relevant genotype/characteristic	Description^*^	Reference
*P. fluorescens* M3A	*nahRG-luxCDABE*	Naphthalene/naphthalene catabolic product detection	Myer, 2013 [13]
*Pseudomonas putida*	*fepA-fes-luxCDABE*	Fe bioavailability	Mioni *et al.* (2003) [9]
*P. fluorescens* HK44	pUTK21 [*luxCDABE*]	Naphthalene catabolism (*Nah*^+^ *Sal*^+^); complements *Nah*^-^ phenotype	King *et al.* (1990) [36]
*P. fluorescens* 5RL	pUTK21 [*luxCDABE*]	Naphthalene catabolism (*Nah*^+^ *Sal*^-^)	King *et al.* (1990) [36]
*Pseudomonas aeruginosa* RLX	*nahRG-luxCDABE*	Naphthalene/naphthalene catabolic product detection	Applegate Strain Collection [13,37]
*S. poona* lux^†^	*kan^R^-luxCDABE*	Constitutive expression of the kanamycin resistance marker	Chen (2008) [38]
*E. coli* K12 lux^†^	pFSP169 [*lac_p_-luxCDABE*]	Constitutive expression from the *lac* promoter	Applegate Strain Collection [6]
*E. coli* O157:H7 lux^†^	pFSP169 [*lac_p_-luxCDABE*]	Constitutive expression from the *lac* promoter	Farris (2007) [39,40]
*V. fischeri* MJ1	Bioluminescence, Wild-type	Constitutive expression	Ruby and Nealson (1976) [41]

*All organisms are grown uninduced and in minimal media unless otherwise noted.

†Are constitutively expressed *lux* cassette.

The solvent effect on the bioreporter organism is summarized in Table 2. When the bioluminescent reporters in the cells were treated with toluene and acetone for 30 min, a significant increase in bioluminescence occurred compared to the control (water). Increases as much as seven- to ninefold over the control were seen in *P. fluorescens* M3A and *Pseudomonas aeruginosa* RLX (*P*<0.05). Comparatively, when the *luxCDABE* cassette was maximally expressed and treated with n-Decanal, bioluminescence was like that of the H_2_O control (*P*>0.05). This interpretation relies on relative changes within the ‘no *n*-decanal’ versus ‘with *n*-decanal’ conditions. Changes in the bioluminescent response to solvents may be explained by the saturation of the bioluminescent biochemical reaction and the absence of substrate limitation. When the organisms were not induced while growing in minimal media, they were affected by sublethal concentrations of solvents due to aldehyde limitation, as previously shown by Heitzer *et al.* [3] [3]. Substrate was neither supplied by the media nor by any biochemical reaction. When solvent-mediated membrane perturbations occur, fatty acids are released into the cell, fueling the bioluminescent response [3]. There is a basal level of expression of the *lux* cassette, even in uninduced states, consequently giving rise to the observed response. Of note, previously examined *lux* expression in *P. fluorescens* HK44 showed that toluene did not induce *lux* mRNA under comparable conditions, even though bioluminescence increased, strongly supporting a mechanism that does not rely on transcriptional induction of the lux cassette [3].

**Table 2.** Bioluminescent detection (relative light units per second) of the solvent effect within several bioluminescent reporter organisms

	No *n-*Decanal			With *n-*Decanal		
	H_2_O (neg. control)	Toluene	Acetone	H_2_O (neg. control)	Toluene	Acetone
*P. fluorescens* M3A	1.00±0.15	9.58±0.07^*^	7.50±0.12^*^	1.00±0.27	1.12±0.04	0.85±0.07
*Pseudomonas putida*	1.00±0.05	1.52±0.06^*^	1.84±0.06^*^	1.00±0.08	1.09±0.02	1.12±0.16
*P. fluorescens* HK44	1.00±0.02	4.04±0.07^*^	1.44±0.04^*^	1.00±0.16	0.80±0.06	0.96±0.14
*P. fluorescens* 5RL	1.00±0.09	4.65±0.11^*^	1.38±0.03^*^	1.00±0.14	0.70±0.20	1.01±0.17
*Pseudomonas aeruginosa* RLX	1.00±0.22	6.97±0.07^*^	7.79±0.07^*^	1.00±0.10	0.80±0.10	1.09±0.21
*S. poona* lux^†^	1.00±0.008	0.98±0.03	1.04±0.08	1.00±0.04	1.09±0.16	1.11±0.12
*E. coli* K12 lux^†^	1.00±0.080	0.97±0.03	1.00±0.04	1.00±0.09	1.02±0.09	1.09±0.10
*E. coli* O157:H7 lux^†^	1.00±0.015	1.01±0.02	0.98±0.04	1.00±0.04	0.97±0.02	1.00±0.04

Values are an average of *n*=3±sd.

*Significantly greater than the control for *α*=0.05.

†Constitutively expressed *lux* cassette.

When light is constitutively expressed, myristic acid recycling occurs (Fig. S1) [2,3]. The addition of toluene and acetone had no measurable effect on bioluminescence from membrane perturbations.

To further determine the effect of substrate limitation, aldehyde (*n-*Decanal) was added to saturate the light system. The bioluminescent response to the tested solvents after addition of *n-*decanal was not significantly greater than the water control for all organisms (*P*>0.05). Since no increase in bioluminescence was noted in response to solvent treatment after aldehyde addition, these data suggest that solvents can influence the bioluminescent response by increasing substrate availability rather than by induction of the *lux* cassette. Indeed, Heitzer [3] demonstrated this response and confirmed that following the addition of toluene to *P. fluorescens* HK44, there was no induction of *lux* mRNA [3].

Using *P. fluorescens* M3A, the bioluminescent response to toluene and acetone treatment in LB and YEPG was observed (Table 3). Media composition, which indicated the available substrates and precursors, was a factor in treating the cells with solvents. There was a significant increase in bioluminescence when the cells were exposed to solvents in a less rich medium compared to the controls (*P*<0.05). Regardless of expression levels, the lack of bioluminescent reaction substrate in the media allowed the cells to respond to solvent-mediated substrate addition. Expression may not play a role because of substrate recycling by induced organisms [3]. Indeed, when comparing induction effects among rich medium-grown organisms, expression of *luxCDABE* provides substrate recycling capabilities, and a solvent effect is seen. Uninduced, rich media organisms cannot recycle substrate, and thus, the reaction is saturated by the substrate available within the media. Uninduced substrate saturation was also demonstrated by the addition of *n-*decanal to the above treatments. In the presence of *n-*decanal, there was no observable bioluminescent increase in uninduced organisms compared to the controls (*P*>0.05).

**Table 3.** Comparison of media and induction effects on the solvent effect within *P. fluorescens* M3A using bioluminescent detection (relative light units per second)

	No *n-*Decanal			With *n-*Decanal		
	H_2_O (neg. control)	Toluene	Acetone	H_2_O (neg. control)	Toluene	Acetone
YEPG – induced	1.00±0.037	1.62±0.134^*^	2.02±0.122^*^	1.00±0.066	1.77±0.119^*^	2.06±0.067^*^
YEPG – uninduced	1.00±0.080	1.53±0.131^*^	2.03±0.145^*^	1.00±0.173	0.92±0.229	1.15±0.110
LB – induced	1.00±0.137	1.76±0.054^*^	2.52±0.043^*^	1.00±0.286	1.60±0.079^*^	2.70±0.097^*^
LB – uninduced	1.00±0.163	0.71±0.240	0.96±0.070	1.00±0.097	0.83±0.174	1.11±0.064

Values are an average of *n*=3±sd.

*Significantly greater than the control for *α*=0.05.

Induction of the *nahRGp-luxCDABE* cassette, inducible by the addition of salicylate.

**Fig. 1.**
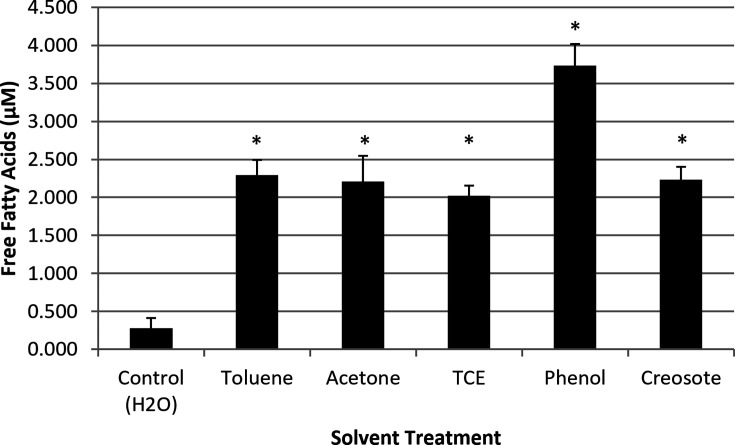
Quantification of solvent effect-mediated fatty acid release within *P. fluorescens* M3A. Each bar represents an average (*n*=3)±sd and applied against a palmitic acid standard curve. Asterisk (*) indicates a result significantly greater than the control for (*α*=0.05).

Previous research has demonstrated that fatty acid concentrations increase within culture media following solvent treatment [3,18] or treatment with carbon nanotubes (CNTs), which induced alterations in the membrane fatty acids of *E. coli* and *Ochrobactrum* sp*.* [19]. To examine the influence of solvents on membrane perturbation and bioluminescent reaction substrate, the release of fatty acids from the membrane in response to solvent treatment was investigated. The measure of free fatty acid release into the extracellular milieu was used as an experimentally accessible proxy for membrane perturbation and liberation of lipid-derived precursors that feed into the aldehyde-generating *luxCDE* pathway. Exposure to various solvents resulted in the release of fatty acids (Fig. 1) into the extracellular milieu, supporting the pronounced bioluminescent response to solvent treatment. Toluene, acetone, TCE and creosote all created similar releases of fatty acids, while phenol demonstrated the greatest release (*P*<0.05). These data are consistent with a model in which membrane damage increases the pool of fatty acid/aldehyde substrates available for the bioluminescent reaction [3,18, 20,23].

### Real-time monitoring of the solvent effect

 Given that the solvent effect was not a result of induction and that membrane perturbations demonstrated fatty acid release into the supernatant, the increase in bioluminescence in response to solvent addition was examined to develop a more detailed kinetic evaluation of the effect. Furthermore, the previous study examining the phenomenon measured the effect after 30 min of treatment [3], necessitating further investigation.

**Fig. 2.**
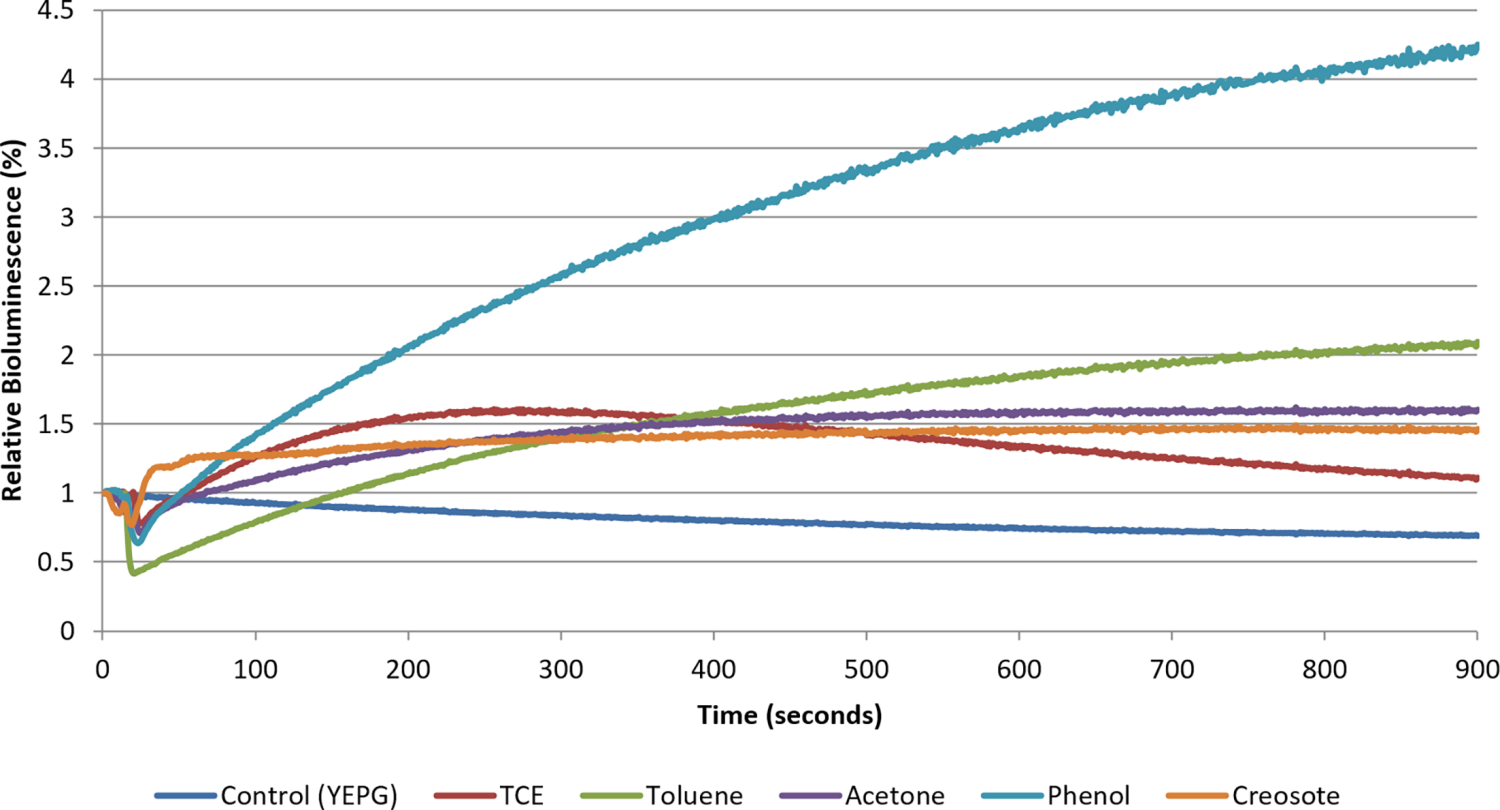
Real-time observation of the solvent effect. Relative bioluminescence of *P. fluorescens* M3A in response to treatment with YEPG (control), TCE (25 p.p.m.), toluene (64 mg l^−1^), acetone (980 mg l^−1^), phenol (25 p.p.m.) and creosote (2000-fold dilution of the water-soluble components). All samples were normalized to the control.

An online, *in situ* monitoring biosensor was developed utilizing *P. fluorescens* M3A (Fig. S2) [13]. In this system, the rate and change of the bioluminescent signal were monitored in real-time in response to the application of solvent. The data in Fig. 2 show that an increase in bioluminescence was detectable in as little as 20 s after the application of the solvent, indicating a rapid method of action that is indicative of the solvent effect. Such a rapid response in bioluminescence following solvent addition is far more consistent with changes in substrate availability and membrane permeability than with transcription/translation-dependent induction, which generally requires minutes to tens of minutes to manifest [24].

Moreover, increases in the bioluminescent signal are substantial compared to the control. All solvents, TCE, toluene, acetone, phenol and creosote, induced a rapid increase in bioluminescence (Fig. 2). Acetone is a common solvent in public use (e.g. degreasing, paint removal, makeup and nail polish remover). Toluene is considered an environmental contaminant and is found as a component of jet fuel [2]. Creosote from beechwood tar is considered a complex solvent in that it consists of a mixture of chemicals, including cresol, phenol and xylenol [15]. Its combined effect on the bioluminescent response to membrane perturbation is evident. Additionally, the response of *P. fluorescens* M3A to creosote (cresol and phenol) and phenol was intriguing.

The use of phenol to determine the phenol coefficient has long been regarded as a measure of the bactericidal activity of a compound [25]. Additionally, cresol has also been noted to be as bactericidal as phenol [17]. Although creosote contains phenolic compounds such as cresol and phenol, its response pattern differed from that of pure phenol. This is likely due to the fact that creosote was applied as a highly diluted water-soluble fraction of a complex mixture, resulting in a substantially lower effective concentration of membrane-active phenolics compared to the defined 25 p.p.m. phenol treatment. Additionally, the heterogeneous composition of creosote may reduce its overall membrane-disruptive activity, as individual components differ in solubility, hydrophobicity and ability to partition into the bacterial membrane [26]. These factors can collectively dampen both fatty acid release and the resulting bioluminescent solvent effect, producing a more moderate response despite the presence of phenolic constituents. The data in Fig. 2 demonstrate that compounds and solvents, typically noted as toxic to micro-organisms and considered environmental contaminants, foster sublethal effects upon the cell at lower concentrations. Such interactions may confound data when utilizing bioluminescent bioreporters to monitor cellular toxicity.

**Fig. 3.**
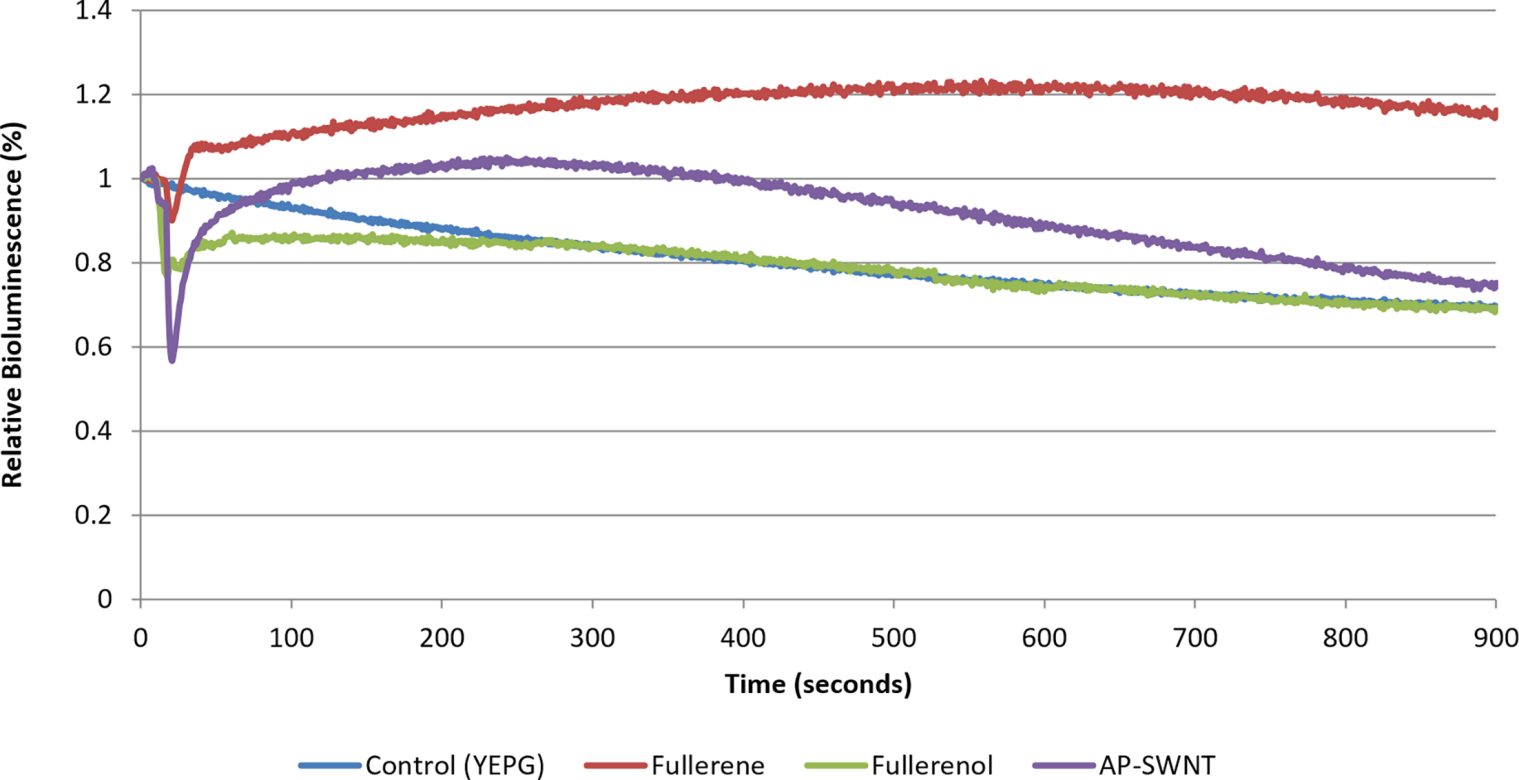
Real-time observation of the solvent effect with nanoparticles. Relative bioluminescence of *P. fluorescens* M3A in response to treatment with YEPG (control), fullerene (50 p.p.m.), fullerenol (25 p.p.m.) and AP-SWNT (3.75 µg ml^−1^). All samples were normalized to the control.

The use of carbon nanoparticles and single-walled carbon nanotubes (SWNTs) in industrial and biomedical applications has grown tremendously over the past decade [27,29]. With the increasing use of such nanoparticles, research addressing their potential environmental release and risk has also increased proportionally. Recently, it has been demonstrated that CNTs can influence membrane fatty acid composition and contribute to toxicity mechanisms [19]. Additionally, recent research utilizing a bioluminescent *E. coli*-O157:H7 strain addressed the response of soil micro-organisms to AP-SWNTs and concluded that SWNTs had toxic effects towards micro-organisms [5]. Accordingly, using the online *in situ* biosensor system, the solvent effect was investigated for other potential membrane perturbation agents. Fig. 3 demonstrates the effect of several carbon nanoparticles on the bioluminescent response of *P. fluorescens* M3A. Eventually falling below the control, AP-SWNTs demonstrated the solvent effect and toxicity, possibly attributable to the cylindrical nature of the particle acting as a pore when interacting with the cell membranes [5]. Fullerene (C_60_) is highly hydrophobic [30]; therefore, it was predicted to interact with cell membranes. As shown in Fig. 3, fullerene caused an increase in bioluminescence compared to the control. Though hydrophobicity is attributed to fullerene partitioning, recent evidence hints at stronger Van der Waals (dispersion) interactions between fullerene and the cell membrane compared to interactions between the fullerene and (bulk) water [31], contributing to the observed solvent effect. Comparatively, when treated with fullerenol (C60(OH)24), a water-soluble fullerene derivative, *P. fluorescens* M3A did not differ in bioluminescence when compared to the control.

Nanoparticles such as fullerenes and AP-SWNTs have optical properties, which implies they absorb a portion of the light emitted [32,33]. Because of this, it is critical to determine if the signal is indicative of light loss or of light absorption. To quantify the light decrease as a result of AP-SWNT and fullerenol addition, a tube-in-tube method was used to create a bioluminescence curve in response to varying concentrations of either agent with *P. fluorescens* M3A (see Fig. S3 for details). This configuration separates the light source and the light quencher from the inside out to observe an alternative to absorbance. Results show that bioluminescence absorbance from *P. fluorescens* M3A by AP SWNTs was negligible at concentrations used in this study (Fig. S3A, *R*²=0.9398, see Supporting Information for details). Alternatively, fullerene and fullerenol experimental values were corrected for light quenching with *P. fluorescens* M3A (Fig. S3 and B-C).

### Membrane toxicity assay

The standard Microtox^®^ assay (AZUR Environmental, Newark, DE) has been regarded as the classical bioluminescent bioreporter assay of acute toxicity effects. The kit utilizes the marine bioluminescent bacterium *V. fischeri* in the basic assay for cellular toxicity. However, the Microtox^®^ assay utilizes agent contact times of up to 30 min and is compared to phenol as a standard. Because certain contaminants will elicit a solvent effect, such as phenol, results may be confounded by increased bioluminescence.

**Fig. 4.**
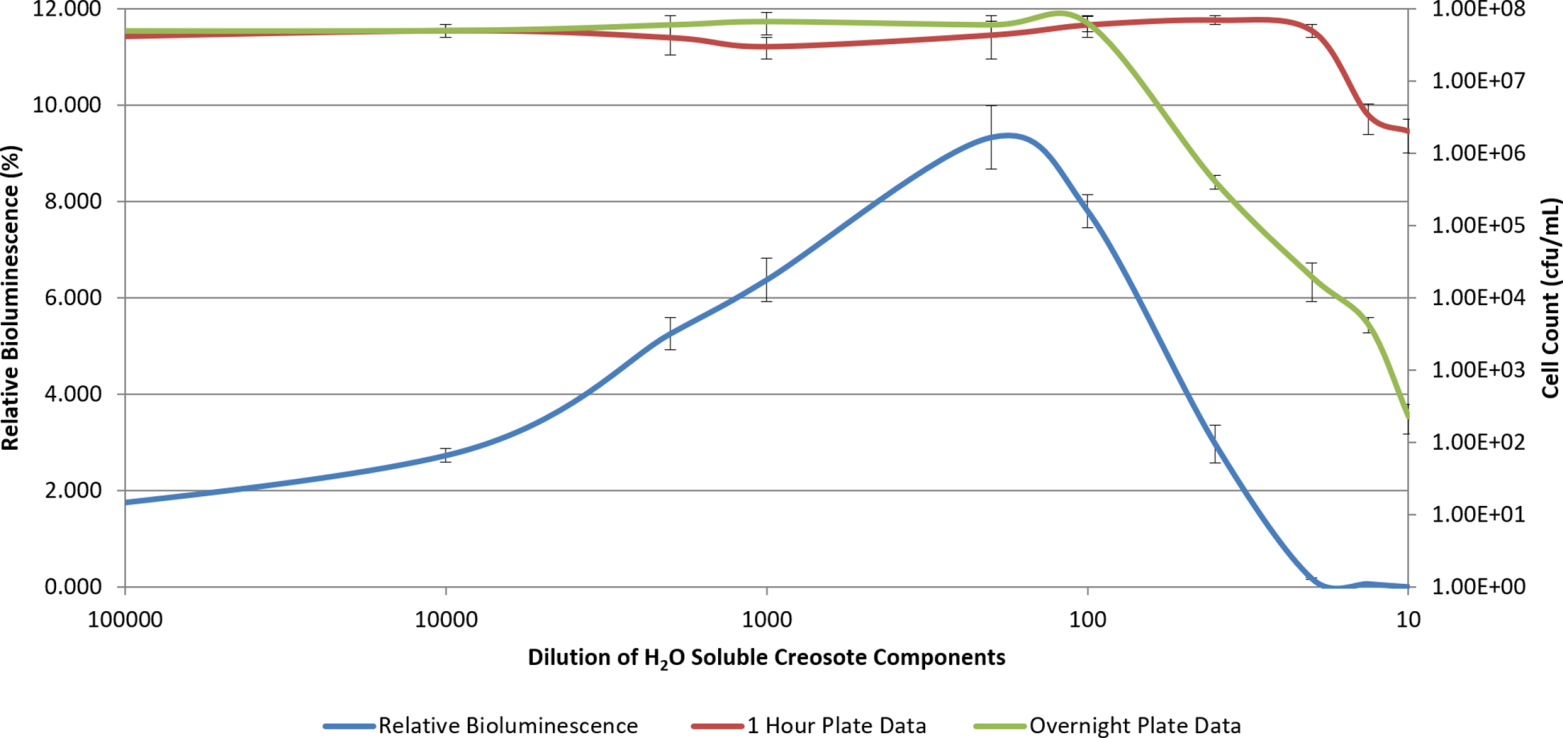
*P. fluorescens* M3A exposed to dilutions of the water-soluble components of creosote (from beechwood tar). Relative bioluminescence (blue) is plotted in tandem with cellular toxicity (c.f.u. ml^−1^) after 1 h (red) and 1 day (green). Each treatment concentration represents an average (*n*=3)±sd.

To examine a potentially more sensitive membrane toxicity assay, *P. fluorescens* M3A was used to determine the toxicity of creosote via membrane damage (Fig. 4). Creosote, being an environmental contaminant of interest in that it consists of multiple solvents (cresol, phenol and xylenol) [15], is a good model for such an assay. Interestingly, bioluminescence increased by up to 9-fold over the control at dilutions of up to 200-fold of the water-soluble components of creosote (*P*<0.05). At fewer dilutions, bioluminescence was quickly diminished, indicating cellular membrane toxicity. When creosote was present at sublethal concentrations (at dilutions of up to 200-fold of the water-soluble components), cell death was not implicated by bioluminescence. In contrast, creosote caused a solvent effect on *P. fluorescens* M3A bioluminescence. Decreases in bioluminescence typically indicate cellular death or impaired metabolism [22]. However, there have been instances in which a decrease in bioluminescence was not indicative of such action. In those cases, hydrostatic pressure-denatured proteins are involved in bioluminescence [34]. At low pressures, the response was reversible, allowing for a restoration of bioluminescence [34]. In this study, such an action was not evident. The membrane toxicity assay was able to detect cellular death ahead of classical plating techniques (Fig. 4). When plated within 1 h of solvent treatment, cellular toxicity was not evident. After 1 day following treatment and plating, cellular toxicity was evident and followed the observed bioluminescent response, indicating the assay’s ability to detect metabolic impairment well before ultimate cell death.

**Fig. 5.**
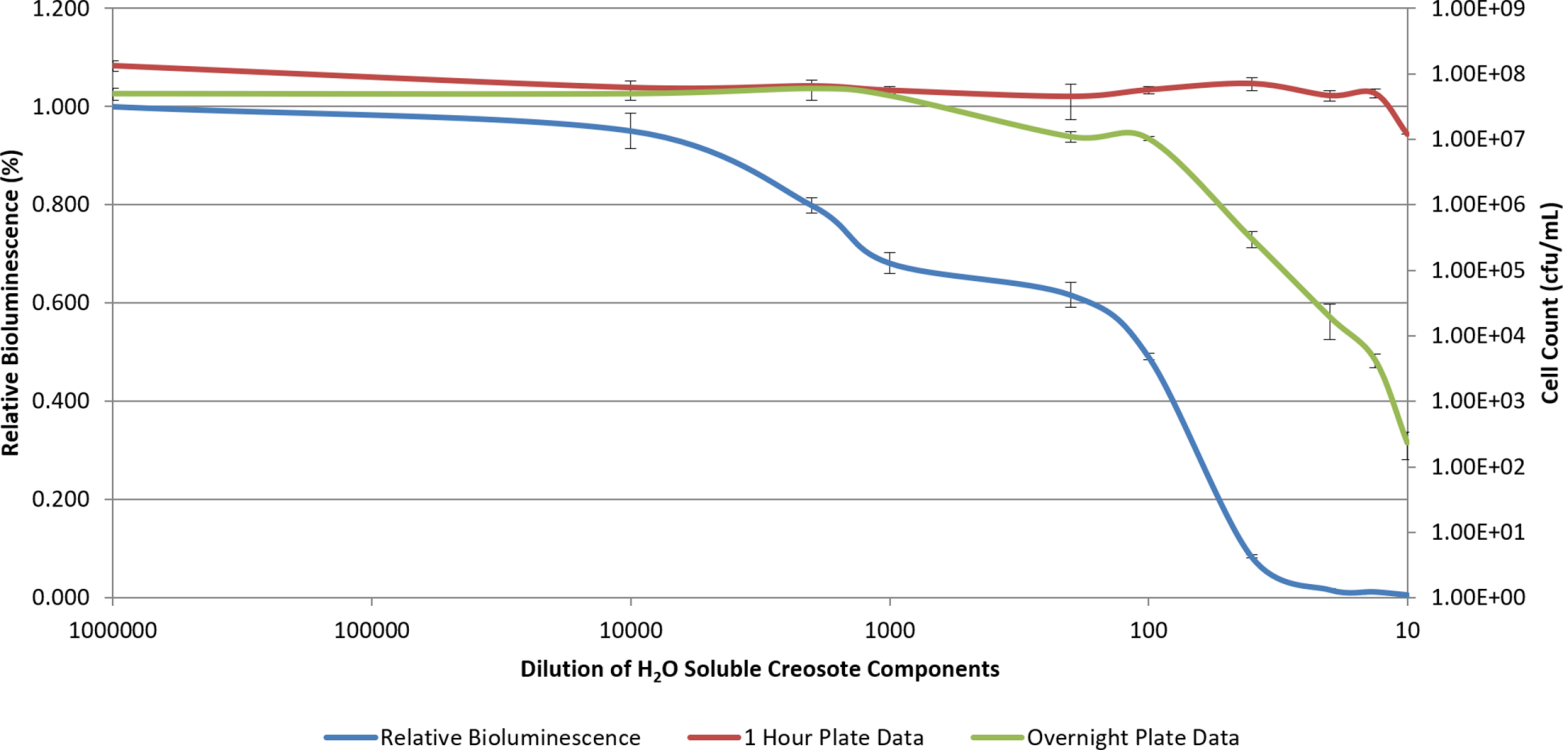
*V. fischeri* MJ1 exposed to dilutions of the water-soluble components of creosote (from beechwood tar). Relative bioluminescence (blue) is plotted in tandem with cellular toxicity (c.f.u. ml^−1^) after 1 h (red) and 1 day (green). Each treatment concentration represents an average (*n*=3)±sd.

**Fig. 6.**
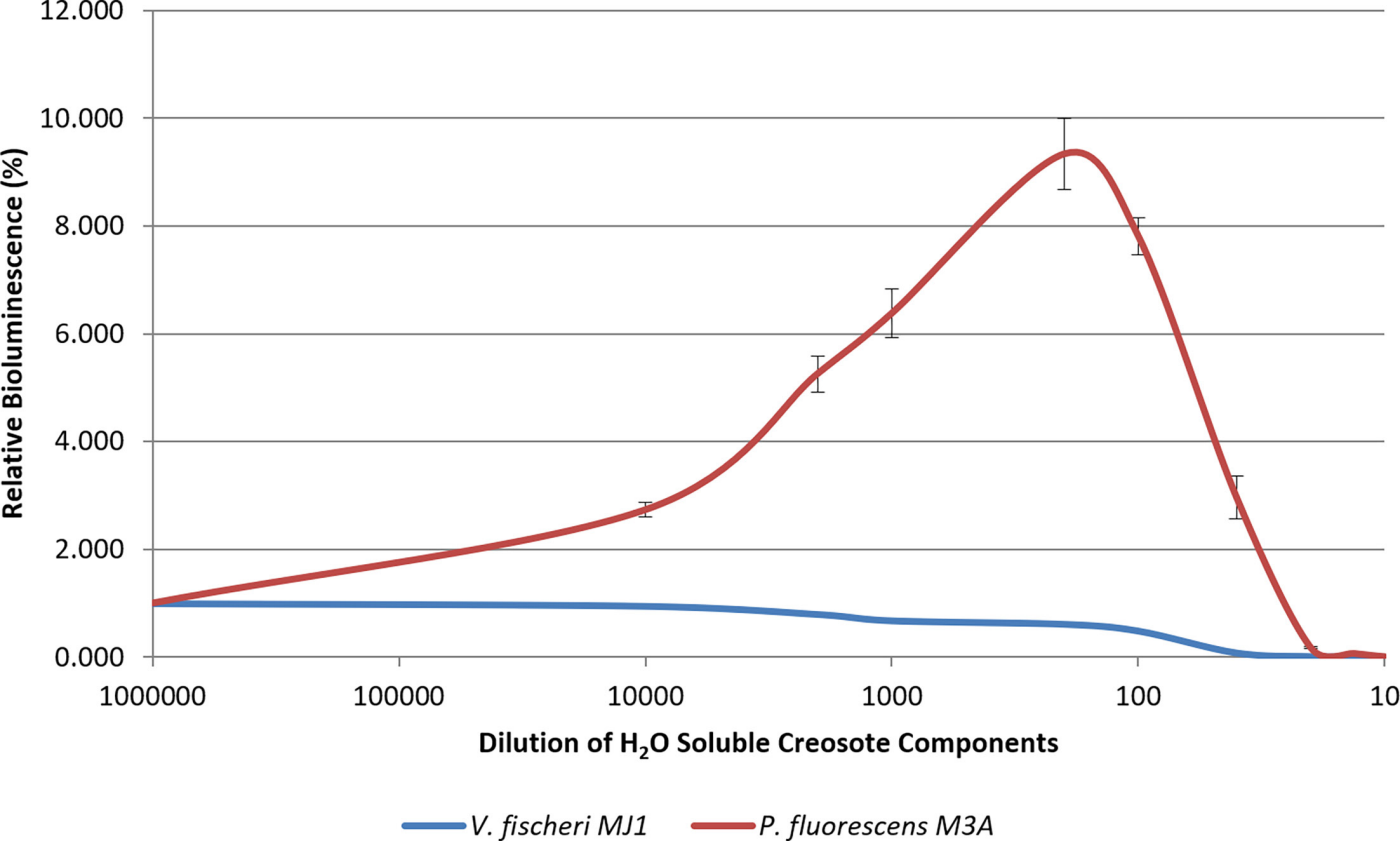
Comparison of bioluminescent toxicity reporting between *V. fischeri* MJ1 and *P. fluorescens* M3A exposed to dilutions of the water-soluble components of creosote (from beechwood tar). Relative bioluminescence of *V. fischeri* MJ1 (blue) and *P. fluorescens* M3A (red) reporting membrane/cellular lethal and sublethal toxicity to the water-soluble components of creosote. Each treatment concentration represents an average (*n*=3)±sd.

Membrane toxicity was also examined using *V. fischeri* MJ1 to determine and compare cellular toxicity. The bioluminescence emitted from *V. fischeri* MJ1 is typically comparatively brighter than that of *P. fluorescens* M3A at the same c.f.u. ml^−1^ [13]. A stronger signal may be desired or warranted in many cases for environmental bioreporters; however, such a strong signal from *V. fischeri* MJ1 may be magnified by quorum sensing, and at greater bacterial load, *V. fischeri* MJ1 cells are constitutively induced [35]. Such expression has been demonstrated not to elicit a strong solvent effect, if any. Data in Fig. 5 show that *V. fischeri* MJ1 did not display a solvent effect with increasing concentrations of creosote-water soluble components. Additionally, bioluminescence decreased even after the addition of 100,000-fold diluted creosote (*P*<005). This represented increased sensitivity to creosote, when compared to *P. fluorescens* M3A, which responded to toxicity only up to 200-fold diluted creosote. However, in the instance of *V. fischeri* MJ1, the initial decrease in bioluminescence did not accurately correlate with toxicity, as shown by cell count data. Toxicity was observed in the 200-fold diluted sample, similar to *P. fluorescens* M3A. Fig. 6 demonstrates the differences in bioluminescence between the two organisms within the assay (*P*<0.05). Although *V. fischeri* MJ1 demonstrated reduced bioluminescence in response to toxicity with increasing creosote concentrations, *P. fluorescens* M3A was more sensitive at detecting sublethal and lethal concentrations, especially with reference to cellular counts. The ability to manipulate *lux* cassette expression, especially in uninduced states, allows *P. fluorescens* M3A to better indicate membrane toxicity. As stated previously, the differences in substrate availability and recycling play a critical role [3]. The constitutive expression of *V. fischeri* MJ1 impedes the ability to determine impending toxicity and cell death from the reduction in bioluminescence. Overall, the magnitude of the solvent effect varied by strain due to inherent differences in membrane composition, known *lux* expression dynamics and substrate limitation. These data indicate that the membrane toxicity assay with *P. fluorescens* M3A is a sensitive method to detect lethal and sublethal effects of environmental contaminants on bacterial bioluminescent bioreporters.

## Conclusions

This study examined and established the solvent effect in many organisms. Data supported that membrane damage or perturbations to the membrane from solvents likely increased the availability of aldehyde precursor levels in the cell. These precursors can supply the required substrates for the bioluminescent reaction (Fig. S1). This suggestion is also supported by data demonstrating the release of fatty acids into the extracellular milieu upon exposure to sublethal concentrations of the tested solvents. Ultimately, this response can confound bioluminescent reporter data, which assumes an inverse correlation between toxic elements and the bioluminescent signal.

Utilizing a biosensor to monitor the solvent effect *in situ*, we demonstrated the rapid action of the solvent effect, which supports previous research indicating that the solvent effect does not involve the induction of *lux* mRNAs [3]. These conclusions regarding *lux* expression are inferred from these physiological patterns and prior data [3], rather than directly measured in the present study. The physical, rapid interactions revealed by the biosensor also demonstrated the shift from sublethal solvent membrane perturbation towards cellular toxicity, dependent on contact time. The membrane toxicity assay helped to reveal the sensitivity of biosensors to toxic agents, as data demonstrated that bioluminescent reporters can indicate cellular death long before it is detectable by traditional culture-based methods. This may be in part due to the connection of the bioluminescent reaction to cellular metabolism, to which the reaction relies heavily on FMNH_2_, O_2_, ATP and substrate [2,22]. When energy and metabolism are altered or impaired, cellular energy pools, redox potential and glucose catabolism are affected, resulting in changes in bioluminescence [2,22, 34]. The bioluminescent reaction is complex, and to better understand the use of bioreporters and the application of biosensors, confounding factors must be accounted for when utilizing bacterial bioluminescence as an indicator of gene expression or cell physiology. Future work quantifying *lux* transcript levels under all solvent conditions would provide further mechanistic confirmation.

## Supplementary material

10.1099/acmi.0.001096.v3 Uncited Supplementary Material 1.

## Abbreviations

AP-SWNTs: as-produced single-walled nanotubes
CNTs: carbon nanotubes
LB: Luria–Bertani
SWNTs: single-walled carbon nanotubes
TCE: trichloroethylene
YEPG: yeast extract peptone glucose
