# Supplementary material for: Application of the solvent effect on bioluminescent reporter bacteria as a real-time membrane toxicity assay
Source: Access Microbiol. 2026 Jan 8;8(1):001096.v3. doi: 10.1099/acmi.0.001096.v3 (PMC12782483; doi:10.1099/acmi.0.001096.v3)

Supporting Information for:

# Application of the Solvent Effect on Bioluminescent Reporter Bacteria as a Real-Time Membrane Toxicity Assay

*Phillip R. Myer* [a*], *Ronald F. Turco* [b], *and Bruce M. Applegate* [c]

[a] Department of Animal Science, University of Tennessee, Knoxville, TN, USA 47996

[b] Department of Agronomy, Purdue University, West Lafayette, IN, USA, 47907

[c] Department of Food Science, Purdue University, West Lafayette, IN, USA, 47907

[*] Corresponding author – pmyer@utk.edu, +1 865-974-3184

List of Content:

Page S2 - **Figure S1.** The Lux Biochemical Pathway.

Page S3 - **Figure S2.** Transducer and components of the *in-situ* system.

Page S4 - **Nanoparticle Light Quenching Analysis**

Page S5 - **Figure S3.** Light absorption by nanoparticles.

**Figure S1.** The Lux Biochemical Pathway. The bioluminescent reaction is catalyzed by the bacterial luciferase gene cassette, *luxCDABE*. The LuxAB heterodimer forms the luciferase which catalyzes the light (490nm) reaction in the presence of oxygen and a reduced riboflavin phosphate (FMNH2) to reduce a long-chain fatty aldehyde (myristyl aldehyde). The aldehyde is synthesized and regenerated by the products of the *luxC*, *luxD*, and *luxE* genes.

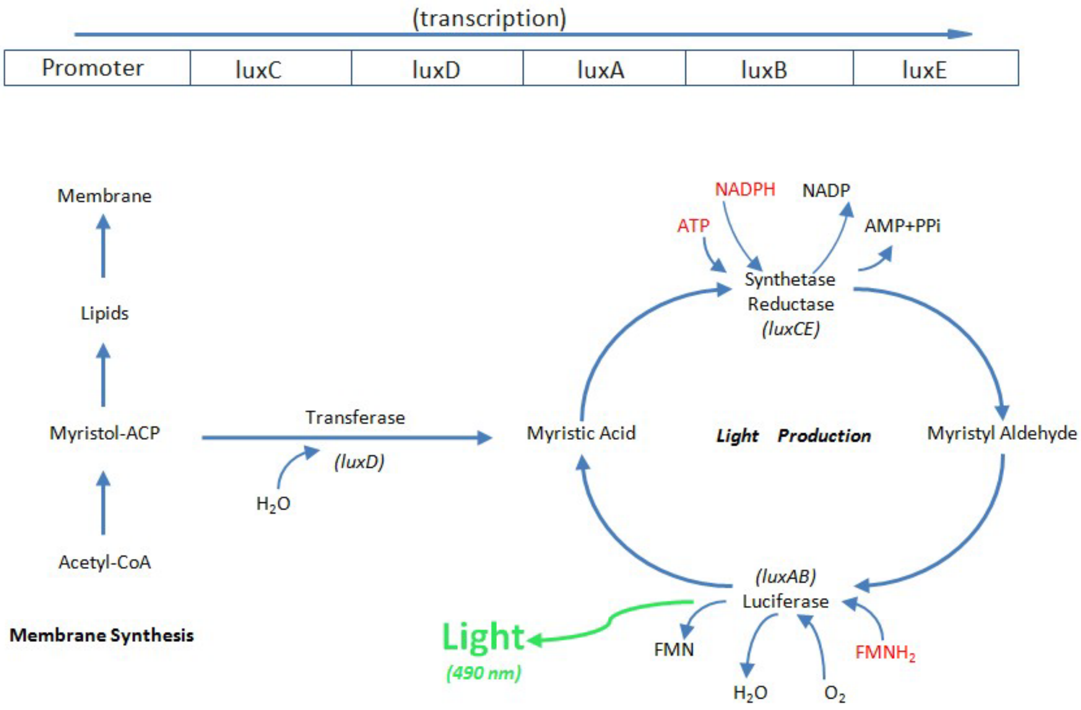

**Figure S2.** Transducer and components of the in-situ system. (A) Whole transducer system placed inside a light tight box. (B) Trace-clean vial and injector set-up. Samples are injected into the system via 3mL syringes + luer lock fittings. Bioluminescence readings were monitored through a light pipe placed in between the scintillation vial and a photomultiplier connected to a PC running customized capturing software.

A

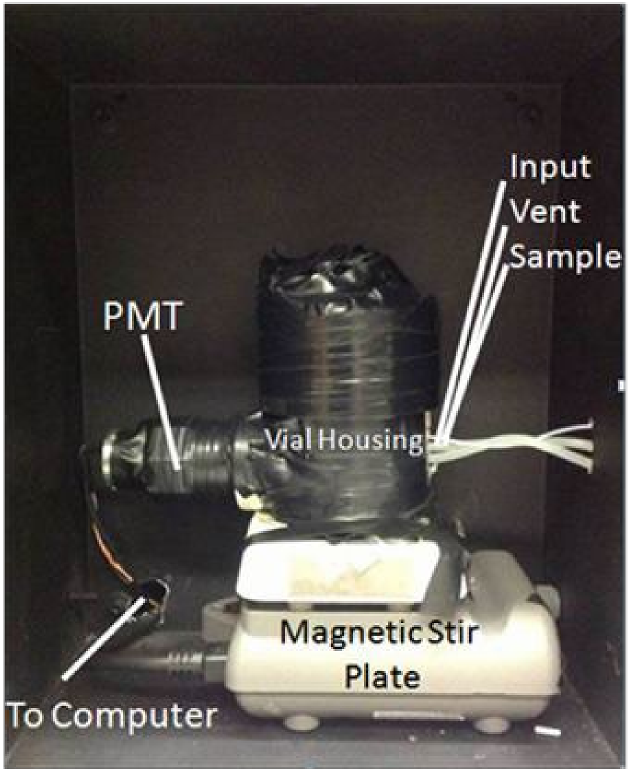


B

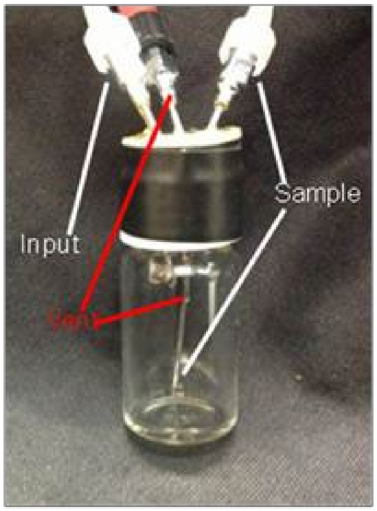

**Nanoparticle Light Quenching Analysis**

Solutions of As-Produced Single-Wall Nanotubes (AP-SWNTs) were prepared in sterile water and serial diluted to obtain 2015, 201.5, 20.15 and 2.015 µg/mL. *P. fluorescens* M3A was grown overnight in YEPG, supplemented with kanamycin (50mg/ml) and salicylate (50mg/ml), and subcultured to an $OD_{600}$ of 0.80. A volume of 0.25 mL *P. fluorescens* M3A was transferred to the inner tube and 1mL of the corresponding AP-SWNT dilution was transferred to the outer tube. Readings were taken from a Zylux luminometer. Light quenching by fullerenol/fullerene was analyzed by varying dilutions of fullerenol/fullerene in mixed solution and as separate solutions. Values were analyzed on a single tube luminometer. The absorbance and transmittance (490nm) of dilutions were documented. Experimental values were then corrected for light quenching effects.

**Figure S3.** Light absorption by nanoparticles. – A tube-in-tube method to determine the quenching of bioluminescence. (A) AP-SWNT. (B) Fullerenol. (C) Fullerene. (D) Tube Configuration

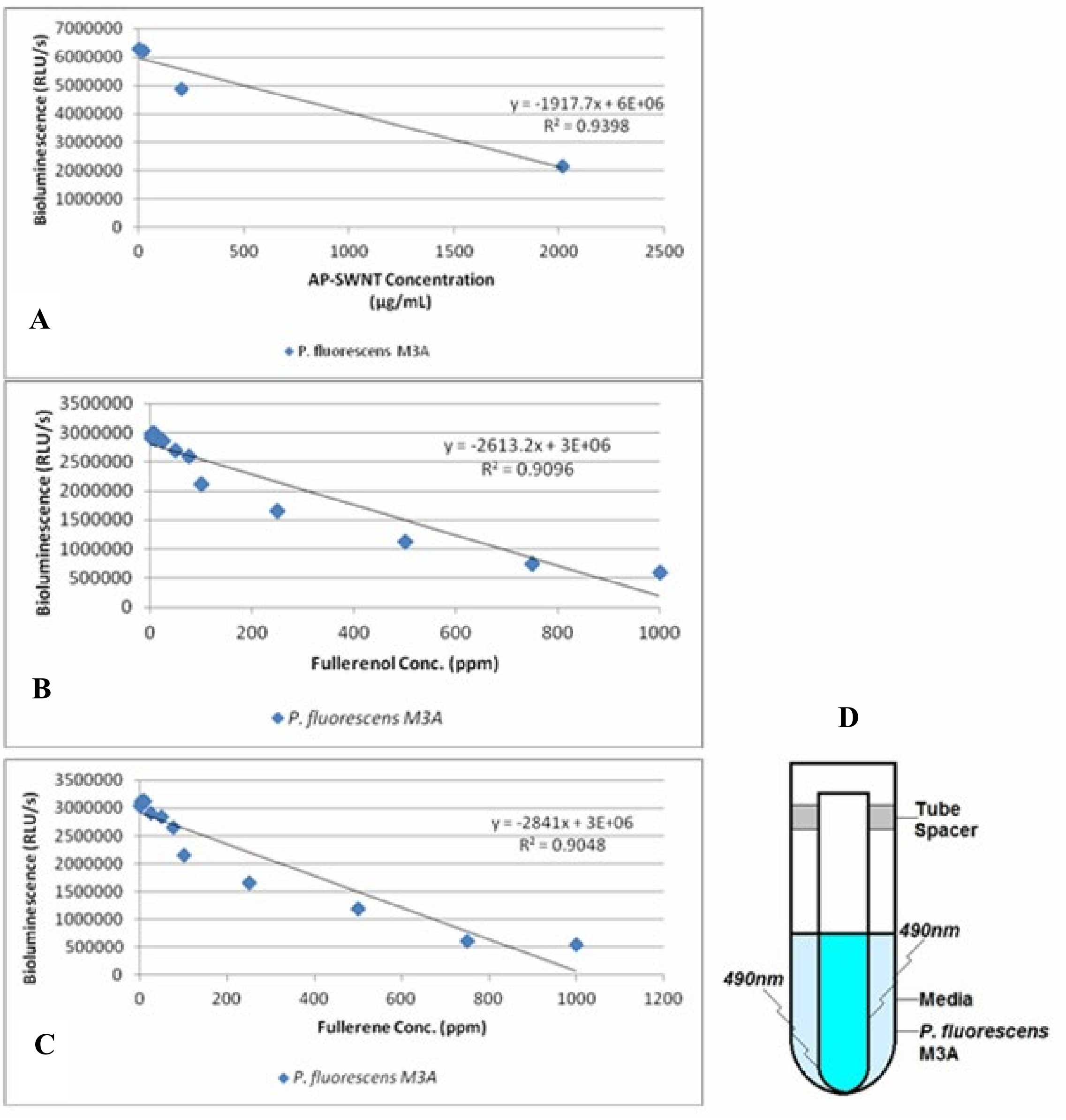

Supplement: Uncited Supplementary Material 1. [file acmi-8-01096-s001.pdf]
